# Impact of *Pneumocystis jirovecii* pneumonia on kidney transplant outcome

**DOI:** 10.1186/s12882-019-1407-x

**Published:** 2019-06-10

**Authors:** Ji Eun Kim, Ahram Han, Hajeong Lee, Jongwon Ha, Yon Su Kim, Seung Seok Han

**Affiliations:** 1 Transplantation Center, Seoul National University Hospital, Seoul, South Korea; 2 Department of Surgery, Seoul National University College of Medicine, Seoul, South Korea; 3 Department of Internal Medicine, Seoul National University College of Medicine, 103 Daehakro, Jongno-gu, Seoul, 03080 South Korea

**Keywords:** Kidney transplantation, Mortality, Outcome, *Pneumocystis jirovecii*, Rejection

## Abstract

**Backgrounds:**

*Pneumocystis jirovecii* pneumonia (PCP) remains an important cause of morbidity and mortality in kidney transplant recipients. While the acute phase toxicity in patients with PCP is well-characterized, there is a lack of data on the effects of PCP on long-term graft outcome.

**Method:**

This retrospective observational study analyzed 1502 adult patients who underwent kidney transplantation at Seoul National University Hospital between 2000 and 2017. After a propensity score matching was performed, the graft and survival outcomes were compared between PCP-negative and PCP-positive groups.

**Results:**

A total of 68 patients (4.5%) developed PCP after transplantation. The multivariable Cox analysis showed that positivity for cytomegalovirus and lack of initial oral antibiotic prophylaxis were risk factors of post-transplant PCP. The PCP-positive group had higher hazard ratios of graft failure [adjusted hazard ratio (HR), 3.1 (1.14–8.26); *P* = 0.027] and mortality [adjusted HR, 11.0 (3.68–32.80); *P* < 0.001] than the PCP-negative group. However, the PCP event was not related with subsequent development of de novo donor-specific antibodies or pathologic findings, such as T-cell or antibody mediated rejection and interstitial fibrosis and tubular atrophy.

**Conclusions:**

PCP is a risk factor of long-term graft failure and mortality, irrespective of rejection. Accordingly, appropriate prophylaxis and treatment is needed to avoid adverse transplant outcomes of PCP.

**Electronic supplementary material:**

The online version of this article (10.1186/s12882-019-1407-x) contains supplementary material, which is available to authorized users.

## Background

With the continued development of immunosuppressive regimens in the past decade, death-censored graft failures have gradually decreased in recipients of both living and deceased kidney recipients [1, 2]. However, the mortality rates in patients with functioning grafts remain unchanged over the past 10 years [1, 2]. More information on the causes of mortality unrelated to graft failure is therefore needed to further improve the survival of transplant recipients.

Infection is an important factor in relation to the risk of death in kidney transplant recipients, and the second most common cause of death after cardiovascular disease in patients with functioning grafts [3–5]. The incidence of most infectious diseases, such as urinary tract infection and sepsis, has been steadily maintained over the past 15 years [6]. Despite its clinical implication of these infectious diseases, there is little data on immunologic outcomes after specific infectious disease in transplant recipients. Further studies of post-transplant infectious events are therefore needed to characterize and ultimately improve survival outcomes.

*Pneumocystis jirovecii* is an opportunistic pathogen that causes severe pulmonary infection in immunocompromized hosts [7]. The incidence of *P. jirovecii* pneumonia (PCP) varies from 0.6 to 14% among kidney transplant recipients without prophylaxis, with a mortality of up to 50% despite aggressive antibiotic therapy [8, 9]. Several studies have investigated the relationship between PCP and mortality [9, 10], but the effect of PCP on graft rejection and overall graft outcomes has been less-well explored. Certain infections such as cytomegalovirus (CMV) and BK virus have demonstrated relationships with acute rejection during the early posttransplant period [11–14]. This is a meaningful clinical issue, given that appropriate infection prophylaxis and treatment regimens could be implemented to address subsequent immunological complications. However, the clinical implications of PCP have not yet been resolved. Herein, we evaluated the impact of PCP on kidney transplant outcomes, including graft failure and rejection.

## Methods

### Study design and subjects

The study design was approved by the institutional review board of Seoul National University Hospital (no. H-1805-173-948) and complied with the Declaration of Helsinki. This retrospective observational study included total 1827 patients who had kidney transplantation at Seoul National University Hospital from January 2000 to December 2017. Patients who were under 18 years old (*n* = 260) or who received simultaneous kidney–pancreas or kidney–liver transplants (*n* = 65) were excluded. Consequently, the remaining 1502 patients were finally included and their data were reviewed. The requirement of informed consent was waived by the board.

### Data collection and definitions

Data from the kidney transplant recipients were collected from the electronic medical records. Demographic characteristics including age, gender, height, weight, and body mass index were collected. Pre-transplant status, including prior history of transplantation, the type and duration of dialysis, history of diabetic nephropathy as a cause of kidney failure, and comorbidities such as hypertension and diabetes, was evaluated.

Results for both recipient and donor ABO blood groups (ABO) and human leukocyte antigen (HLA) typing were collected to evaluate ABO compatibility and the number of HLA mismatches. Immunosuppressive therapy was determined, including induction therapy with basiliximab or anti-thymocyte globulin, and calcineurin inhibitors for maintenance therapy. A combination of steroids, mycophenolic acid, and calcineurin inhibitors was the standard maintenance immunosuppressive regimen in our center. PCP was defined as the presence of findings suspicious of PCP detected by a radiologist on chest computed tomography combined with PCP positivity on polymerase chain reaction or direct immunofluorescence stain of sputum or bronchoalveolar lavage fluid. Initial use of oral prophylactic antibiotics was defined as a prescription of trimethoprim/sulfamethoxazole for more than 4 weeks during the first month after kidney transplantation. There were no cases with pentamidin or atovaquon for prophylaxis. CMV positivity was defined as > 20 copies/ml on polymerase chain reaction or positive result on viral culture, according to the definition of CMV infection [15].

### Transplant outcomes

The primary outcome was death-censored graft failure defined as a return to dialysis or kidney re-transplantation. The secondary outcome was all-cause mortality, based on data obtained from the National Database of Statistics Korea. All patients were followed until graft failure or March 2018. Data on biopsy-proven acute T-cell-mediated rejection, acute antibody-mediated rejection, and interstitial fibrosis and tubular atrophy were also collected. Protocol biopsies were performed at zero-time (postreperfusion), on the 10th day and 1 year after transplantation, and every year thereafter. Additional kidney biopsies were performed if graft function deteriorated or any suspicious symptoms or signs of rejection were observed. All pathologic findings were examined by a nephropathologist. The development of de novo donor-specific antibody (DSA) was defined as any newly developed anti-HLA class I or II antibody.

### Statistical analysis

All statistical analyses were performed using Stata software (version 15.1, StataCorp, College Station, TX, USA). Data are expressed as mean ± standard deviation for continuous variables or counts with percentages for categorical variables. Among the baseline characteristics, categorical variables were compared using χ^2^ and Fisher’s exact tests and continuous variables were compared using Student’s *t*-test. Risk factors for PCP occurrence were analyzed using univariable and multivariable Cox proportional hazard models.

Propensity score matching was performed to account for the imbalance in baseline characteristics between the PCP-positive and -negative groups. Scores were created with matching variables including age, gender, donor type, type of pre-transplant dialysis, hypertension, the usage of prophylactic antibiotics, and CMV positivity which had *P* values under 0.1 in multivariable Cox analysis for the risk of PCP. Then, the cases were matched on propensity score in a 1:2 block, using a nearest neighbor matching algorithm with replacement, using the statistical package psmatch2. Following propensity score matching, graft survival, overall patient survival, risk of rejection and development of DSA were analyzed using univariable and multivariable Cox proportional hazard models. Because PCP infection was a time-dependent covariant in the Cox model, we used the stssplit function in Stata to split the time at which PCP occurred. The proportionality assumption was checked for proportional hazard Cox regression. Survival curves were drawn using the Kaplan–Meier method, with comparisons between groups carried out using the log-rank test. A *P* value < 0.05 was considered to indicate statistical significance.

## Results

### Baseline characteristics and risk factor of PCP

Table 1 shows the demographic and clinical characteristics of the total study subjects, according to the PCP status. The median duration of follow-up was 6.2 years (interquartile range, 3.0–9.6 years; maximum 18.3 years). Of the 1502 patients, 68 (4.5%) experienced PCP after kidney transplantation, with an infection rate of 6.8 cases per 1000 person-years. The median time to the development of PCP was 5.2 months (interquartile range, 3.9–10.0 months), and 79.4% of cases developed during the first year after transplantation. There were significant differences between PCP-positive and -negative patients with respect to gender, type of pre-transplant dialysis, ABO-incompatibility, desensitization therapy, induction regimen, hypertension and CMV positivity. After adjustment for multiple covariates, CMV positivity and the non-use of oral prophylactic antibiotics were associated with an increased risk of PCP (Table 2).

**Table 1:** Baseline characteristics of total study subjects

Variables	Total (*n* = 1502)	PCP negative (*n* = 1434)	PCP positive (*n* = 68)	*P*
Age (years)	45.3 ± 13.0	45.1 ± 13.0	48.3 ± 14.4	0.053
Female (%)	39.1	39.9	23.5	0.007
Body mass index (kg/m^2^)	22.5 ± 3.7	22.5 ± 3.8	22.6 ± 2.6	0.969
Donor type (%)				0.124
Living	67.4	67.8	58.8	
Deceased	32.6	32.2	41.2	0.391
Standard criteria donor	25.4	25.2	29.4	
Expanded criteria donor	7.2	7.0	11.8	
Pre-transplant dialysis (%)				0.005
Preemptive	15.6	15.3	22.1	
Hemodialysis	65.2	66.0	47.1	
Peritoneal dialysis	19.2	18.7	30.9	
Duration of dialysis (months)	40.1 ± 47.7	40.0 ± 47.5	42.9 ± 51.9	0.650
Re-transplantation	7.2	7.2	7.4	0.958
ABO-incompatible (%)	7.4	6.8	19.1	< 0.001
HLA mismatch (numbers)	3.1 ± 1.7	3.1 ± 1.7	3.1 ± 1.7	0.983
HLA-A	0.9 ± 0.7	0.9 ± 0.7	1.0 ± 0.8	0.344
HLA-B	1.2 ± 0.7	1.2 ± 0.7	1.2 ± 0.8	0.861
HLA-DR	1.0 ± 0.7	1.0 ± 0.7	0.9 ± 0.7	0.291
Calcineurin inhibitor (%)				0.260
None	3.1	3.0	5.9	
Cyclosporine	13.0	13.2	8.8	
Tacrolimus	83.9	83.8	85.3	
mTOR inhibitor (%)	1.9	1.9	2.9	0.535
Induction regimen (%)				0.022
None	19.0	19.6	7.4	
Basliximab	79.6	78.9	92.6	
Antithymocyte globulin	1.4	1.5	0	
Desensitization (%)	11.5	10.9	22.1	0.005
Diabetes mellitus (%)	19.0	18.9	22.1	0.517
Hypertension (%)	89.9	89.5	98.5	0.016
DMN for kidney failure (%)	16.4	16.3	19.1	0.543
Positivity for CMV (%)	21.4	19.5	60.3	< 0.001
Oral prophylactic antibiotics (%)	18.8	19.2	10.3	0.067

Comparisons were evaluated between PCP-negative and PCP-positive groups

Abbreviations: *PCP Pneumocystis jirovecii* pneumonia, *HLA* human leukocyte antigen, *DMN* diabetic nephropathy, *mTOR* mammalian target of rapamycin, *CMV* cytomegalovirus

**Table 2:** Risk factors for PCP occurrence after kidney transplantation

Variables	Univariable		Multivariable*	
	HR (95% CI)	*P*	HR (95% CI)	*P*
Age	1.02 (1.00–1.04)	0.015	stratified	
Female	0.46 (0.27–0.81)	0.007	0.56 (0.30–1.06)	0.074
Body mass index	1.01 (0.95–1.08)	0.761		
Donor type				
Living donor	1 (reference)		1 (reference)	
Standard criteria donor	1.43 (0.83–2.46)	0.194	1.89 (0.91–3.94)	0.090
Expanded criteria donor	2.22 (1.03–4.77)	0.041	1.14 (0.37–3.50)	0.818
Pre-transplant dialysis				
Preemptive	1 (reference)		1 (reference)	
Hemodialysis	0.51 (0.28–0.95)	0.033	0.42 (0.19–0.89)	0.023
Peritoneal dialysis	1.10 (0.57–2.13)	0.786	0.91 (0.39–2.11)	0.829
Duration of dialysis	1.00 (1.00–1.01)	0.464		
Re-transplantation	1.03 (0.41–2.56)	0.948		
ABO-incompatible	3.75 (2.13–6.93)	< 0.001	1.60 (0.38–6.77)	0.521
HLA mismatch (numbers)	1.02 (0.88–1.17)	0.834		
Calcineurin inhibitor				
None	1 (reference)		1 (reference)	
Cyclosporine	0.27 (0.08–0.97)	0.045	0.34 (0.07–1.65)	0.179
Tacrolimus	0.52 (0.19–1.42)	0.202	0.41 (0.11–1.54)	0.185
Induction regimen			Stratified	
None	1 (reference)			
Basiliximab	4.43 (1.73–11.32)	0.002		
Antithymocyte globulin	NE	NE		
Desensitization	2.67 (1.49–4.76)	0.001	2.67 (0.66–10.76)	0.167
Diabetes mellitus	1.31 (0.74–2.33)	0.354		
Hypertension	7.87 (1.09–56.73)	0.041	6.59 (0.80–54.14)	0.080
DMN for kidney failure	1.32 (0.72–2.41)	0.373		
Positivity of CMV	6.41 (3.93–10.44)	< 0.001	4.48 (2.58–7.79)	< 0.001
Oral prophylactic antibiotics	0.54 (0.25–1.19)	0.129	0.35 (0.15–0.82)	0.016

*Adjusted for gender, donor type, ABO compatibility, desensitization, hypertension, positivity of CMV and oral prophylactic antibiotics. Because age and induction regimen violated the proportionality assumption, multivariable Cox model was stratified by age and induction regimen

Abbreviations: *PCP Pneumocystis jirovecii* pneumonia, *HR* hazard ratio, *CI* confidence interval, *HLA* human leukocyte antigen, *NE* not estimable, *DMN* diabetic nephropathy, *CMV* cytomegalovirus

### PCP and transplant outcomes

**Fig. 1:**
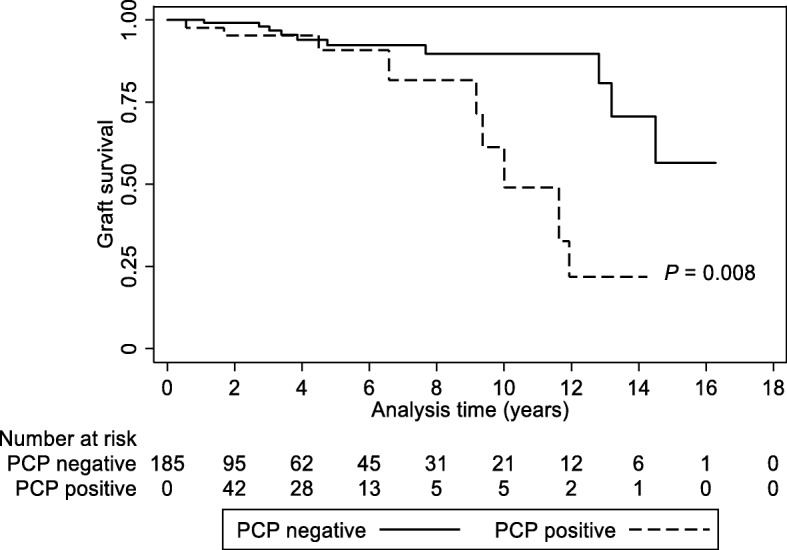
Overall graft survival curves in the PCP-positive and -negative patients. *P* value was obtained using the log-rank test. Dashed line, PCP-positive; solid line, PCP-negative

We performed propensity score matching to mitigate the difference in baseline characteristics between the PCP-positive and -negative groups. Table 3 shows the baseline characteristics of the two groups of patients after propensity score matching. Among 68 PCP-positive recipients, 9 (13.2%) patients developed death-censored graft failure. Figure 1 shows the Kaplan-Meier curves for death-censored graft survival, and the curves were separated by the presence of PCP (*P* = 0.008). Cox regression analysis considering PCP as a time-dependent variable was subsequently performed. Both univariable and multivariable analyses showed a significant relationship between PCP and graft failure, with HRs of 3.34 (1.31–8.56) (*P* = 0.012) and 3.33 (1.30–8.53) (*P* = 0.012), respectively. Although the acute T cell-mediated and antibody-mediated rejection episodes or other immunological findings such as interstitial fibrosis and tubular atrophy and de novo DSA were additionally adjusted, the PCP-positive recipients had a higher risk of death-censored graft failure than the PCP-negative recipients (adjusted HR, 3.06 [1.14–8.26]); *P* = 0.027).

**Table 3:** Baseline characteristics after propensity score matching

Variables	PCP negative (*n* = 118)	PCP positive (*n* = 68)	*P*
Age (years)	46.7 ± 14.6	48.3 ± 14.4	0.504
Female (%)	21.8	23.5	0.792
Body mass index (kg/m^2^)	22.5 ± 3.4	22.6 ± 2.6	0.509
Donor type (%)			0.447
Living	64.5	58.8	
Deceased	35.5	41.2	0.846
Standard criteria donor	24.5	29.4	
Expanded criteria donor	10.9	11.8	
Pre-transplant dialysis (%)			0.102
Preemptive	15.3	22.1	
Hemodialysis	68.6	47.1	
Peritoneal dialysis	16.1	30.9	
Duration of dialysis (months)	47.4 ± 52.1	44.1 ± 54.0	0.704
Re-transplantation	6.8	7.4	0.883
ABO-incompatible (%)	7.6	19.1	0.019
HLA mismatch (numbers)	3.1 ± 1.5	3.1 ± 1.7	0.915
Calcineurin inhibitor (%)			0.349
None	2.5	5.9	
Cyclosporine	13.6	8.8	
Tacrolimus	83.9	85.3	
Induction regimen (%)			0.108
None	15.3	7.4	
Basliximab	82.2	92.6	
Antithymocyte globulin	2.5	0	
Desensitization (%)	11.0	22.1	0.043
Diabetes mellitus (%)	20.3	22.1	0.781
Hypertension (%)	94.1	98.5	0.149
DMN for kidney failure (%)	19.5	19.1	0.950
Positivity for CMV (%)	57.6	60.3	0.722
Oral prophylactic antibiotics (%)	14.4	10.3	0.420
Acute T cell-mediated rejection (%)	66.9	61.8	0.475
Acute antibody-mediated rejection (%)	9.3	11.8	0.596
Interstitial fibrosis and tubular atrophy (%)	37.3	42.6	0.471
De novo donor-specific antibody (%)	12.7	11.8	0.850

Comparisons were evaluated between PCP-negative and PCP-positive groups

Abbreviations: *PCP Pneumocystis jirovecii* pneumonia, *HLA* human leukocyte antigen, *DMN* diabetic nephropathy, *CMV* cytomegalovirus

The clinical information on the PCP-positive patients with graft failure (*n* = 9) are shown in Additional file 1: Table S1. All the patients were male and had no history of prophylactic antibiotics usage after transplantation. During the admission period, 8 of 9 patients (88.9%) experienced acute kidney injury according to the Kidney Disease Improving Global Outcomes criteria [16], and 4 of them (50%) did not have recovered their graft functions at discharge despite the recovery from PCP. To determine the cause of the elevated risk of graft failure, we evaluated the effect of PCP on the risk of subsequent occurrence of rejection, interstitial fibrosis and tubular atrophy, and de novo DSAs. However, these outcomes did not differ between PCP-positive and –negative groups (Table 4). The results suggest that different graft failure rates might not be attributable to the conventional immunological transplant episodes, but might be related with non-immunological factors such as concurrent acute kidney injury.

**Table 4:** Risk of rejection and development of de novo donor-specific antibody according to the occurrence of PCP

Outcomes	Univariable		Multivariable*	
	HR (95% CI)	*P*	HR (95% CI)	*P*
T-cell mediated rejection	0.49 (0.19–1.26)	0.140	0.50 (0.19–1.28)	0.148
Antibody mediated rejection	0.70 (0.15–3.27)	0.651	0.66 (0.14–3.08)	0.597
Interstitial fibrosis and tubular atrophy	1.51 (0.72–3.15)	0.274	1.62 (0.77–3.41)	0.204
De novo donor specific antibody	0.87 (0.28–2.66)	0.801	0.88 (0.29–2.70)	0.821

*Adjusted for variables which had *P* values less than 0.1 in Table 3

Abbreviations: *HR* hazard ratio, *CI* confidence interval

**Fig. 2:**
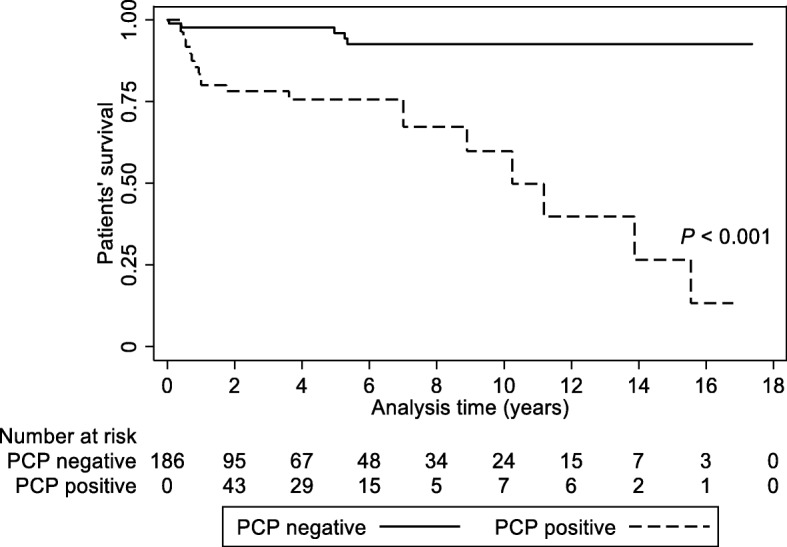
Patient survival curves in the PCP-positive and -negative patients. *P* value was obtained using the log-rank test. Dashed line, PCP-positive; solid line, PCP-negative

During the follow-up period, 11 patients (16.2%) and 13 patients (11.0%) died in the PCP-positive and -negative groups, respectively. The 28 PCP-positive patients had concurrent other infectious diseases, and 8 patients died (28.6%). All-cause mortality was higher in the PCP-positive group than in the PCP-negative group (Fig. 2). The PCP-positive group had a higher risk of mortality [adjusted HR, 10.99 (3.68–32.80); *P* < 0.001] than the PCP-negative group.

Additional file 1 Table S2 lists the PCP-positive patients who died. Nine of Eleven patients (81.8%) died within 3 months after the occurrence of PCP. The most common cause of death was acute respiratory distress syndrome related with PCP.

## Discussion

*Pneumocystis jirovecii* is an ascomycetous fungus that causes opportunistic infections, and its life cycle remains unknown because it cannot be consistently cultured [17, 18]. Furthermore, the epidemiology of human PCP is unclear yet [17, 19]. Due to little knowledge about *Pneumocystis jirovecii*, it is necessary to investigate whether the long term effect of PCP on allografts is due to the infection alone or with chronic inflammatory process and other unknown mechanisms. Although PCP is relatively common after kidney transplantation, its clinical implications, especially in relation to graft outcomes, have not been fully evaluated. The results of the present study demonstrated that male gender and CMV positivity were risk factors associated with PCP, and the use of oral prophylactic antibiotics seemed to prevent the risk of PCP. The occurrence of PCP increased the risk of long-term graft failure; however, this relationship was not dependent of rejection, interstitial fibrosis and tubular atrophy, or de novo DSAs. Because PCP was associated with both graft failure and overall mortality, intensive treatment and prophylaxis is recommended after transplantation.

Previous studies have shown that CMV infection increases a risk of PCP [20–22], as supported by the present results. This may be because CMV might modify the host immune response, leading to immune suppression [23, 24]. CMV infection affects the T cell compartment and accelerates aging of T cells [25]. T-cells, especially CD4 T cells, are important factors affecting the vulnerability to and resolution of PCP [26], and alteration of the T cell response by CMV might aggravate pulmonary impairment during PCP activation [27].

Pre-transplant dialysis was newly identified as a protective factor of PCP. None of previous studies have included this factor in the analysis models. Uremic condition is linked to altered immunological responses [28]. Accordingly, appropriate pre-transplant resolution of uremia would decrease the risk of post-transplant PCP. However, a long-term pre-transplant dialysis negatively affects the host immune status [29]. Additionally, other detailed information such as the dose or adequacy of dialysis which the present study did not obtain could function as an interacting factor. Further studies are needed to determine the underlying mechanisms of above relationship.

The occurrence of PCP itself did not affect the risk of following development of acute rejection and de novo DSA production. Nevertheless, occurrence of PCP was significantly associated with overall graft failure. As has been previously demonstrated, viral infections such as CMV, BK virus, and hepatitis C virus, and certain bacterial infections such as *Pseudomonas aeruginosa*, affect allograft dysfunction by modulating non-immunological factors such as hemodynamic change in addition to immunological factors [30, 31]. In our results, PCP did not increase the risk of any rejection after infection, suggesting that non-immunological factors may be the cause underlying the observations. Certain PCP-positive patients had concurrent acute kidney injury and half of them did not achieve the recovery from this condition, which might leave the grafts with non-immunological damages. However, through non-immunological pathways, infections may also induce allograft injury by stimulating the production of proinflammatory cytokines such as interleukin-1, interleukin-6, and interleukin-8 [32]. These proinflammatory cytokines are known to be upregulated in patients with PCP [26].

Mortality risk was elevated in patients with PCP. Nine out of eleven (81.8%) patients died within 3 months after diagnosis of PCP infection. The causes of death in these patients were either PCP itself or the other infection superimposed on PCP. Thus, considering both the significant effect of PCP on mortality and the negative effect on graft outcome over long-term period, prophylaxis against PCP may be strongly recommended. According to the Kidney Disease Improving Global Outcomes guideline, it is recommended that all recipients receive prophylaxis against PCP for 3–6 months after transplantation [33]. However, definitive guidelines on the duration and dosage of PCP prophylaxis are not available and more research is needed to determine the appropriate approach [33].

Although our data are informative, there are some limitations. First, it was a retrospective study, making it difficult to demonstrate cause and effect definitively. Nevertheless, the study achieved its primary purpose by demonstrating the relationship between PCP and transplant outcomes. Second, the current definition of prophylactic antibiotics was 1 month, which was shorter than the guideline recommendation (i.e., 3–6 months), which might increase overall risk of PCP in study subjects. The duration of antibiotic prophylaxis (i.e., more than 4 weeks) might differ between centers, which could also alter the risk of PCP or other transplant outcomes. However, this did not hinder the study purpose on the relationship between PCP and transplant outcomes. Lastly, the present observational study design could not determine the mechanisms underlying the risk and subsequent effect of PCP.

## Conclusion

Risk of PCP is aggravated in the kidney transplant cases with male gender, positivity for CMV, and non-use of oral prophylactic antibiotics. PCP significantly increases both the risks of mortality and graft failure. Accordingly, robust prophylaxis may be needed to prevent PCP and subsequent graft failure. The present results will be the basis of the future clinical trials on the use of prophylaxis in kidney transplant recipients.

## Additional files

Additional file 1: **Table S1.** Information on the patients who had graft loss after the occurrence of *Pneumocystis jirovecii* pneumonia. **Table S2.** Information on the patients who died after the diagnosis of *Pneumocystis jirovecii* pneumonia. (DOCX 20 kb)

## Data Availability

The datasets used and/or analyzed during the current study are available from the corresponding author on reasonable request.

## Abbreviations

CI: Confidence interval
CKD: Chronic kidney disease
CMV: Cytomegalovirus
DMN: Diabetic nephropathy
DSA: Donor-specific antibody
HLA: Human leukocyte antigen
HR: Hazard ratio
PCP: *Pneumocystis jirovecii* pneumonia
