# Supplementary material for: Impact of Pneumocystis jirovecii pneumonia on kidney transplant outcome
Source: BMC Nephrol. 2019 Jun 10;20:212. doi: 10.1186/s12882-019-1407-x (PMC6558901; doi:10.1186/s12882-019-1407-x)
Supplement: Supplementary file 1 — Table S1. Information on the patients who had graft loss after the occurrence of Pneumocystis jirovecii pneumonia. Table S2. Information on the patients who died after the diagnosis of Pneumocystis jirovecii pneumonia. (DOCX 20 kb) [file 12882_2019_1407_MOESM1_ESM.docx]

Table S1. Information on the patients who had graft loss after the occurrence of *Pneumocystis jirovecii* pneumonia

| No. | TPL to PCP  (months) | PCP to GF  (months) | Prophylactic antibiotics | Creatinine (mg/dL) | | | †eGFR (ml/min/1.73 m^2^) | |
| --- | --- | --- | --- | --- | --- | --- | --- | --- |
|  |  |  |  | *Before PCP | Peak Cr during hospitalization | **After PCP | *Before PCP | **After PCP |
| #1 | 142.9 | 2.3 | No | 4.57 | 8.68 | 6.01 | 14.7 | 10.7 |
| #2 | 106.4 | 35.1 | No | 2.18 | 4.28 | 2.08 | 33.3 | 35.2 |
| #3 | 67.7 | 46.3 | No | 2.5 | 3.09 | 2.39 | 33.5 | 29.8 |
| #4 | 107.9 | 3.7 | No | 3.02 | 3.23 | 3.24 | 23.5 | 21.5 |
| #5 | 102.3 | 19.4 | No | 4.46 | 5.05 | 4.15 | 15.1 | 17.2 |
| #6 | 58.0 | 22.1 | No | 2.67 | 5.04 | 3.63 | 24.2 | 16.9 |
| #7 | 3.7 | 51.0 | No | 1.62 | 5.02 | 2.14 | 45.1 | 32.7 |
| #8 | 20.4 | 0.1 | No | 2.71 | 4.62 | 3.26 | 24.0 | 19.4 |
| #9 | 5.3 | 1.4 | No | 1.60 | 3.86 | 1.45 | 45.0 | 50.4 |

* The last outpatient based laboratory result before admission for PCP.

** The first outpatient based laboratory result after discharge.

†eGFR was calculated by The Modification of Diet in Renal Disease study equation

Abbreviations: TPL, transplantation; PCP, *Pneumocystis jirovecii* pneumonia; GF, graft failure; Cr, creatinine; eGFR, estimated glomerular filtration rate.

Table S2. Information on the patients who died after the diagnosis of *Pneumocystis jirovecii* pneumonia

| No. | TPL to PCP  (months) | PCP to death  (months) | Prophylactic antibiotics | Concurrent infection | Cause of death |
| --- | --- | --- | --- | --- | --- |
| #1 | 2.7 | 121.9 | No | None | Biliary sepsis with Enterococcus faecium infection |
| #2 | 134.7 | 1.4 | No | CMV | PCP with septic shock |
| #3 | 3.2 | 2.5 | No | *Staphylococcus aureus*,  *Aspergillus fumigatus* | PCP with ARDS |
| #4 | 9.0 | 35.0 | No | Influenza A virus | Diffuse alveolar hemorrhage, pneumonia with no pathogen |
| #5 | 20.4 | 0.9 | No | *Acinetobacter baumannii* | PCP with ARDS |
| #6 | 11.6 | 0.6 | No | *Acinetobacter baumannii*  *Klebsiella pneumoniae* | PCP with septic shock |
| #7 | 7.2 | 1.6 | No | None | PCP with ARDS |
| #8 | 9.5 | 1.8 | No | *Acinetobacter baumannii* | PCP with ARDS |
| #9 | 5.3 | 3.0 | No | *Aspergillus fumigatus*,  CMV | PCP with pulmonary aspergillosis |
| #10 | 6.1 | 0.5 | No | *Acinetobacter baumannii* | PCP with ARDS |
| #11 | 4.3 | 0.6 | No | None | PCP with ARDS |

Abbreviations: PCP, *Pneumocystis jirovecii* pneumonia; TPL, transplantation; CMV, cytomegalovirus; ARDS, acute respiratory distress syndrome.
